# A Continuum-Tensegrity Computational Model for Chondrocyte Biomechanics in AFM Indentation and Micropipette Aspiration

**DOI:** 10.1007/s10439-022-03011-1

**Published:** 2022-07-25

**Authors:** Alessandro Arduino, Sofia Pettenuzzo, Alice Berardo, Valentina A. Salomoni, Carmelo Majorana, Emanuele Luigi Carniel

**Affiliations:** 1 Department of Civil, Environmental and Architectural Engineering, University of Padova, Padua, Italy; 2 Department of Biomedical Sciences, University of Padova, Padua, Italy; 3 Department of Management and Engineering, University of Padua, Padua, Italy; 4 Department of Industrial Engineering, University of Padova, Padua, Italy

**Keywords:** Cell mechanics, Finite element model, AFM indentation, Micropipette aspiration, Tensegrity

## Abstract

Mechanical stimuli are fundamental in the development of organs and tissues, their growth, regeneration or disease. They influence the biochemical signals produced by the cells, and, consequently, the development and spreading of a disease. Moreover, tumour cells are usually characterized by a decrease in the cell mechanical properties that may be directly linked to their metastatic potential. Thus, recently, the experimental and computational study of cell biomechanics is facing a growing interest. Various experimental approaches have been implemented to describe the passive response of cells; however, cell variability and complex experimental procedures may affect the obtained mechanical properties. For this reason, in-silico computational models have been developed through the years, to overcome such limitations, while proposing valuable tools to understand cell mechanical behaviour. This being the case, we propose a combined continuous-tensegrity finite element (FE) model to analyse the mechanical response of a cell and its subcomponents, observing how every part contributes to the overall mechanical behaviour. We modelled both Atomic Force Microscopy (AFM) indentation and micropipette aspiration techniques, as common mechanical tests for cells and elucidated also the role of cell cytoplasm and cytoskeleton in the global cell mechanical response.

## Introduction

Apart from genes and chemical factors, mechanical stimuli are important regulators for the development of organs and tissues, their growth and connected processes such as remodelling, regeneration or disease (References 20, 21, 29 to cite a few). This strong influence is due to the fact that cells can sense and respond to mechanical signals, by converting them into biochemical responses (this process is called mechanotransduction, firstly coined in 1998^6^). Moreover, external mechanical forces control the shape, type and function of a living cell by altering the internal balance of the cell itself,^7^ thus, modifying the internal prestress of cell main subcomponents (the cytoskeleton, the membrane, the cytoplasm and the nucleus) and consequently influencing the biochemical signals produced by the cell.^21,31,40^

The evidence that biochemistry and signals transmission can be modified by cell mechanics variations is also strictly connected to apparently unrelated diseases manifestation, where an abnormal mechanotransduction, sometimes combined with alterations of the Extra Cellular Matrix (ECM) mechanical properties, influences a disease development and spreading.^21,29^ In particular, for oncological pathologies, tumour cells are usually characterized by a decrease in the mechanical properties that provides the cell with a higher deformation and mobility, thus suggesting that cell mechanics can be directly linked also to its metastatic potential.^9,16,20,21,42^ For this reason, the study of cell mechanics and related properties is facing a growing interest in the last years, from both an experimental and a computational point of view.

Various experimental approaches have been used to describe the passive response of cells,^18^ as the AFM indentation,^8–11^ which is able to locally stress the cell with compression forces, thanks to a microscale cantilever with a spherical tip, or the Micropipette Aspiration (MPA),^12,15,17,27,34,38,39^ by means of microscale pipette tips that can apply a negative pressure to the cell surface, thus exciting the cell with tensile stresses. Other techniques, as the Magnetic Tweezers (MT), are more interested in determining the mechanical properties of cell subcomponents such as the cytoskeleton.^4^ However, experimental cell response measurements are usually characterized by a huge variability due to cell phenotypes and types,^8,11,12,26,28,33,34,38^ shape,^8^ source^9,26,33^ and aging.^30^ This being the case, it is quite difficult to fully describe the mechanical behaviour of a cell by means of in *in-vivo* tests, and even more difficult to extract the role of each subcomponents.

For this reason, *in-silico* computational models have been developed through the years, to overcome such limitations and uncertainties, while proposing a valuable tool to understand cell mechanical behaviour.^1–3,5,24,28,32,41^ Some of these models describe the cell as an homogeneous viscoelastic material,^1–3,41^ others specify the main subcomponents adopting a tensegrity structure to model the cytoskeleton,^5,24,25,28^ as stated and assessed in past works.^22,23,40^

Computational models usually aim at describing cells undergoing AFM indentation, accounting for cell mechanics variability^3^ or the effect of an altered cytoskeleton to predict the mechanics of cancer cells,^24^ as well as MPA, to understand the influence of the pipette shape and the material properties.^1,15,41^

For this purpose, thanks to a computational approach, we used finite elements to analyse the mechanical response of a cell experiencing both AFM indentation and MPA techniques, in order to: (i) develop a homogeneous model that is able to catch the global viscoelastic response of a cell subjected to both compression and tensile mechanical stimuli; (ii) propose a combined continuous-tensegrity model to observe how each subcomponent contributes to the overall mechanical behaviour; (iii) compare the two models to elucidate the influence of the cytoplasm and the cytoskeleton, by varying their mechanical properties. We firstly set both the homogeneous and the continuum-tensegrity models for the description of the AFM indentation tests, then we validated their applicability with the MPA simulations, highlighting from these two types of tests some useful insights on the subcellular components roles.

## Materials and Methods

### Finite Element Models of the Cell and Its Subcomponents

Three-dimensional (3D) models of a cell were realized with the finite element software Abaqus Explicit 2019 (Dassault Systemes Simulia Corp., Providence, RI).

**Figure 1:**
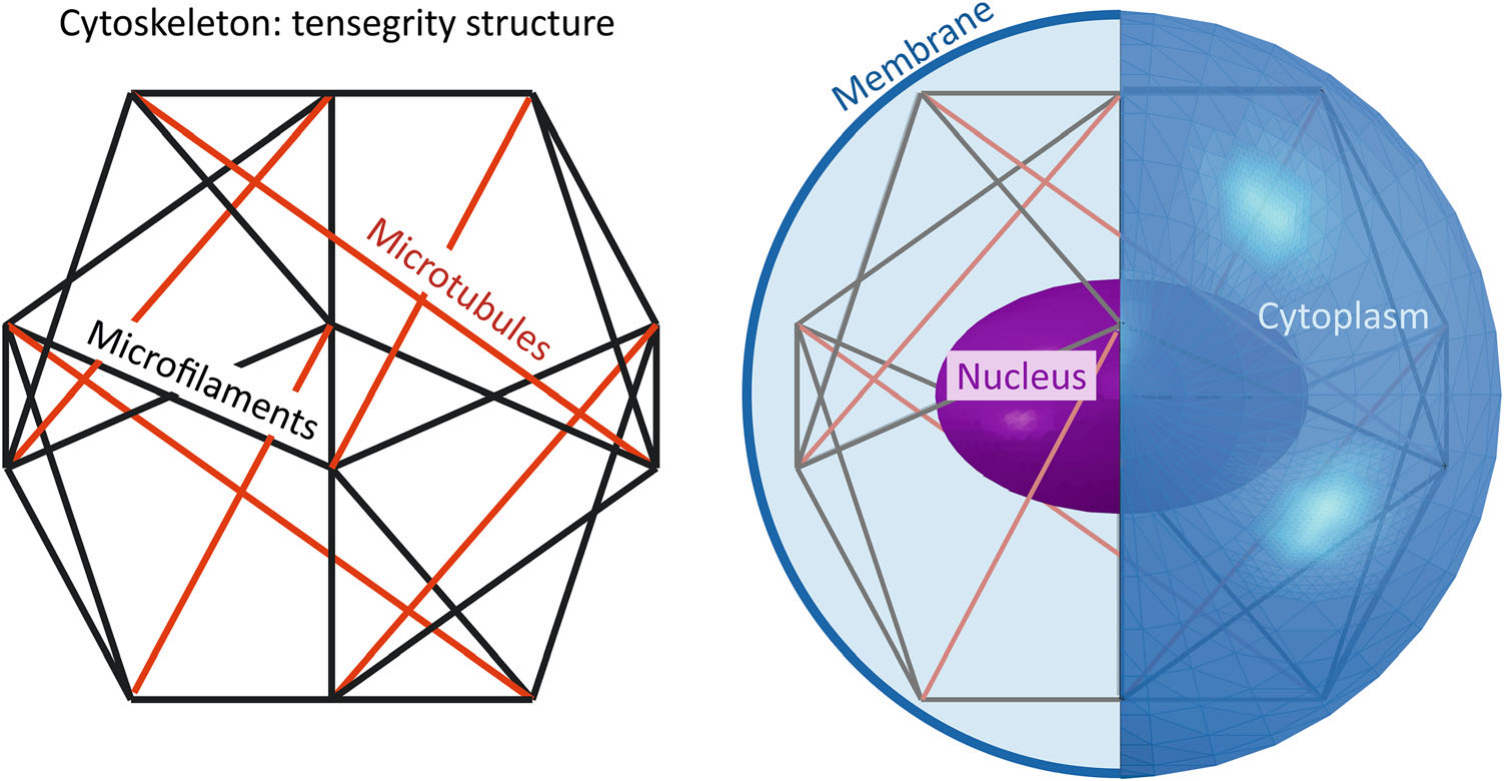
Continuum-tensegrity model of the cell, where the lattice represents the cytoskeleton (with microfilaments in black and microtubules in red), while nucleus, membrane and cytoplasm are described as homogeneous materials.

The model simulated a 16 μm diameter cell (with reference to Ref. 28), composed of all those features that mainly contribute to the cell mechanics, such as the cytoskeleton (microfilaments and microtubules), the cytoplasm, the cell membrane and the nucleus (Fig. 1). A homogeneous continuous model (CM) and a combined continuous-tensegrity model (CMT) were developed to analyse the mechanical response of a cell undergoing AFM indentation and aspiration through micropipette.

In the CM the cell was represented with a 3D solid-sphere composed only by the cytoplasm, discretized by means of about 100.000 linear hexahedral elements, 110.000 nodes and 360.000 degrees of freedom. Within the CTM, the nucleus was represented, with reference to the literature 28, as an ellipsoid (major axis 8 μm and minor axis 5 μm) while the membrane was modelled with a shell part with a constant thickness of 6 nm. In this case the cytoskeleton was described with a tensegrity structure of six compression bearing struts and twenty-four tensional cables that are able to mimic the real behaviour of the microtubules and the microfilaments, respectively.^5,22,23,28^ The connection between each strut and cable created twelve common nodes, representing the ‘receptor’ sites where actin filaments clustered at adhesion complexes. Regarding their geometry, the microtubules had a cross-sectional area of 190 nm^2^, while the microfilaments were thinner, with an area of 18 nm^2^.^28^ The CTM was discretized with a number of linear hexahedral elements between 70.000 and 400.000 depending on the model complexity, about 35.000 linear quadrilateral elements for cell membrane and 3000-linear truss elements for the cytoskeleton. In the model, the nodes of the membrane were coincident with the underlying cytoplasm and with cytoskeletal receptor sites, and tie constraints were assigned to cell subcomponents. The total number of nodes was within the range 105.000–440.000 resulting in 370.000–1.500.000 degrees of freedom.

Even if the model has the potential to mimic all kinds of cells, this work primarily focused on chondrocytes (specialized cells present in the cartilage) and could be addressed to chondrosarcoma cells (malignant tumour cells that origin from chondrocytes), thanks to a larger availability of data in the literature with respect to other cells.

### Finite Element Model of Atomic Force Microscope (AFM) Indentation

The Atomic Force Microscope indentation was performed on a rounded cell adherent to a rigid substrate, where cell height and contact radius with the substrate were assumed with reference to Ref. 28, thus about 14 μm and 6 μm, respectively. A spherical rigid body simulated the cantilever tip of the AFM. Different analyses were performed with 5 μm and 10 μm tip diameter sizes, to observe possible changes induced by the indenter size in the load–displacement response of both the CM and CTM, such as non-linear contribution of the cell subcomponents. The contact between the cell and the indenter was assumed to be frictionless, for the tangential behaviour, and hard contact type, for the normal behaviour. The bottom nodes, at the cell-substrate interface, were constrained in all three translational degrees of freedom. These constrained points mimicked the focal adhesion sites in cells adherent to a substrate, thus implies that the substrate is adopted as rigid. In some recent works the influence of a solid substrate has been studied, especially when a large probe indents a spread cell, thus some bottom-effects corrections have been proposed.^13,14^ However, thanks to the comparison with the Hertz model of the contact between two spheres and the models outputs, we noticed no undesired effects due to the applied boundary conditions, probably also because we are adopting an almost spherical configuration of the cell, instead of a spread one.

**Figure 2:**
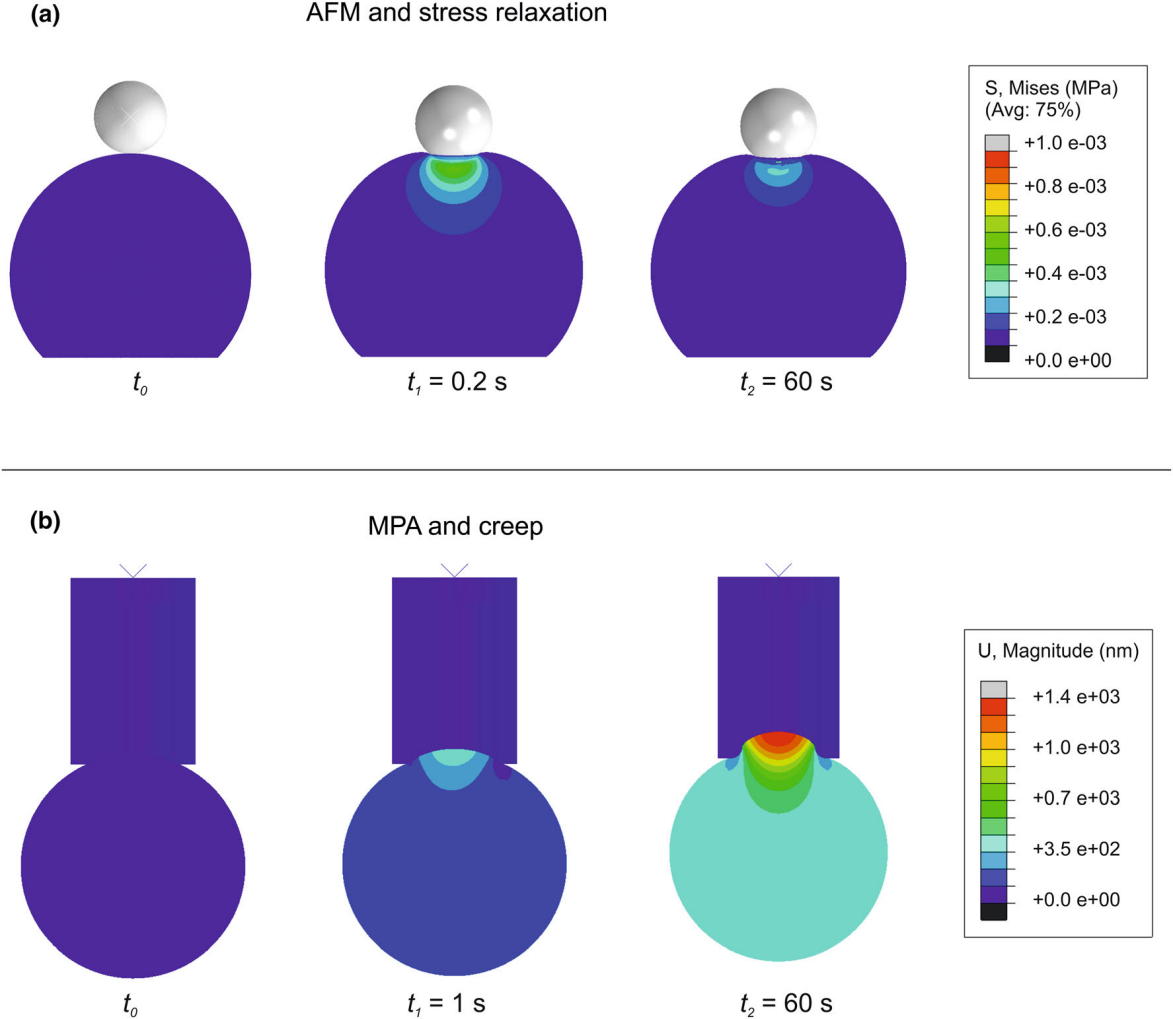
Analyses steps. (a) AFM and stress relaxation: at time *t*_*0*_ the rigid sphere indents the cell. After an average time of *t*_*1*_ the maximum displacement is reached and stress relaxation begins. Few variations of stress distributions are visible after time *t*_*2*_. Von Mises stresses distribution is reported with colormap and graduated scale (in MPa). (b) MPA and creep: at time *t*_*0*_ the rigid micropipette is in contact with the cell. After *t*_*1*_ the maximum negative pressure is applied and kept constant, to observe the creep behaviour of the cell until time *t*_*2*_. Total cell displacement is reported with colormap and graduated scale (in nm).

The analyses were performed with a two-step dynamic explicit simulation. Similarly to experimental protocols reported in literature,^10^ during the first step, a 1500 nm displacement was applied by the spherical indenter to the top of the cell; then this loading phase was followed by a relaxation one in which the cantilever was held in place for up to 60 s (Fig. 2a). In this way, stress relaxation tests were performed on the central region of the cell using a 9.5 μm/s approach velocity.^8^

### Finite Element Model of Micropipette Aspiration (MPA)

The Micropipette Aspiration tests simulated the interaction with a rounded visco-hyperelastic cell and a round rigid cylindrical pipette with a smooth-edged mouth and 900 nm-round fillet. In this case, several simulations were realized, considering different ratios between the cell and the micropipette radii, more precisely with ratio values of 1.5, 2, 3, 4 and 5.5. The contact between the cell and the micropipette was assumed to be frictionless (tangential behaviour), and hard contact type (for the normal behaviour).

The Micropipette Aspiration tests were performed with a two-time step dynamic explicit simulation, similarly to the AFM indentation. During the first step, lasting 1 s, the cell was exposed to a fluid negative pressure variation $- \Delta$P, within the micropipette. After this phase, the creep response of the cell was analysed by keeping a constant $- \Delta$P and measuring the projection length *L*_*P*_ of the cell inside the pipette for up to 60 s (Fig. 2b).

Since there was a concentration of the deformation gradient near the pipette fillet, a dense mesh in the upper part of the cell was adopted, for both the cytoplasm and the plasma membrane.

### Mechanical Properties of Cell Subcomponents

Both the CM and the CTM were tested with viscoelastic and visco-hyperelastic material properties. The Neo-Hookean formulation was used for the hyperelastic material model. The mechanical properties adopted in this study are reported in Tables 1 and 2, with reference to the several analysed configurations.

**Table 1:** Mechanical properties adopted for the continuum cell model.

	Cytoplasm						
	*E*_*el*_ (MPa)	*E*_*R*_ (MPa)	*τ*_*σ*_ (s)	*τ*_*ε*_ (s)	*ν* (–)	*C*_*10*_ (MPa)	*D*_*1*_ (MPa^−1^)
Viscoelastic model	1.28E−03	4.50E−04	19.7	9.5	0.37	–	–
Visco-hyperelastic model	–	–	19.7	9.5	0.37	2.33E-4	1220.66

**Figure 3:**
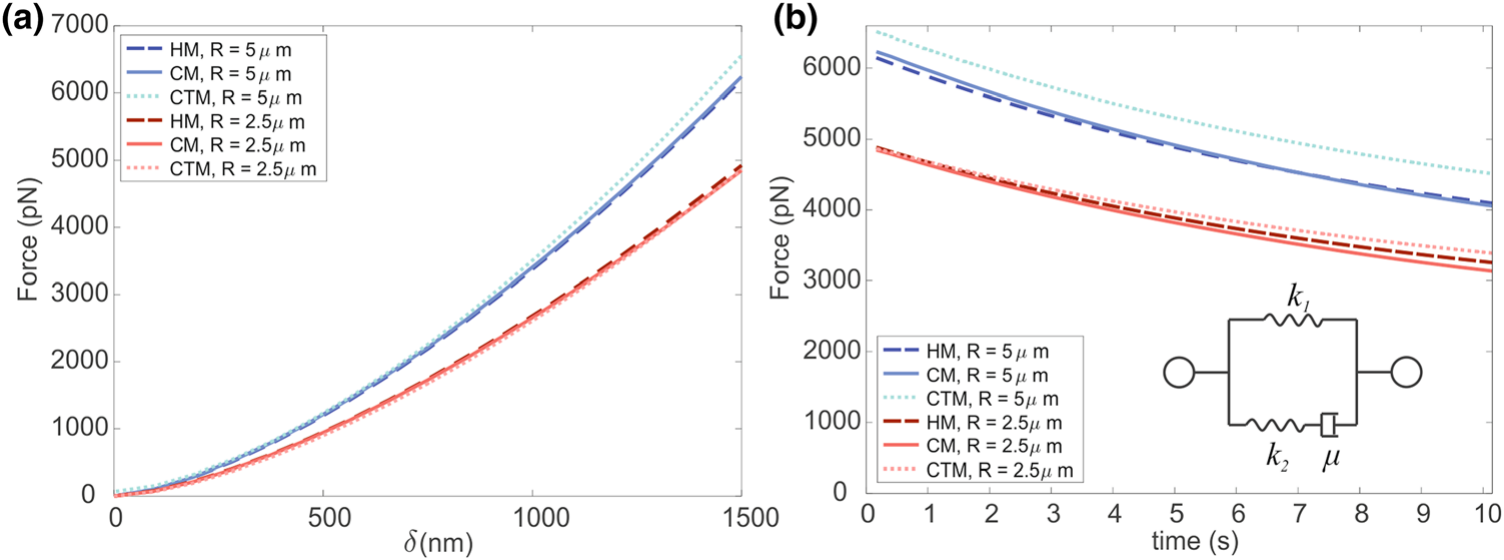
Comparison between the Hertz analytical model (HM, dashed blue and red lines), the homogeneous continuum model with hyperelastic formulation (CM, full lines) and the continuum-tensegrity model (CTM, dotted lines) during AFM loading phase (a) and the stress relaxation phase (b). Two indenter sizes (*R* = 2.5 μm and 5 μm) were analysed with an indentation length of 1.5 μm. Mechanical parameter that were used for both the loading and stress-relaxation phases are reported in Tables 1 and 2.

*E*_*el*_ is the Young’s modulus assuming a linear elastic formulation for the loading phase; *E*_*R*_ is the relaxed modulus (with reference to^8^), which corresponds to *k*_*1*_ of the Standard Linear Solid model (Fig. 3b); *τ*_*σ*_ time of relaxation of deformation under constant load; *τ*_*ε*_ time of relaxation of load under constant deformation; *ν* Poisson’s coefficient; *C*_*10*_ and *D*_*1*_ are the parameters of the corresponding Neo-Hookean model

**Table 2:** Mechanical properties adopted for the continuum-tensegrity cell model.

	Continuoum-tensegrity visco-hyperelastic model						
	*E*_*el*_ (MPa)	*E*_*R*_ (MPa)	*τ*_*σ*_ (s)	*τ*_*ε*_ (s)	*ν* (−)	*C*_*10*_ (MPa)	*D*_*1*_ (MPa^−1^)
Cytoplasm	1.28E−03	4.50E−04	19.7	9.5	0.37	2.33E−04	1.22E+03
Microtubules	1.53E+04	–	–	–	0.38	2.78E+03	9.39E−05
Microfilaments	3.32E+04	–	–	–	0.38	6.02E+03	4.33E−05
Cell membrane	1.28E−02	–	–	–	0.3	2.46E−03	1.88E+02
Nucleus	5.11E−03	–	–	–	0.37	9.33E−04	3.05E+02

Parameters notation is the same as reported in Table 1

Both AFM indentation and MPA analyses lasted between 1 and 72 h, depending on the model complexity and the number of required steps, running contemporary on 20 threads of a High-Performance Computing Server Fujitsu Primergy RX4770 equipped with four Intel Xeon E7 8890 v4 processors, 512 GB RAM and SSD HD.

## Results

### AFM Indentation: Loading Phase and Stress Relaxation Behaviour

A typical representation of the AFM indentation experiments is usually performed by reporting the force-displacements curves obtained at the cantilever tip. A good analytical model usually employed in this case is the Hertz contact model,^10^ reported in Eq. (1), which describes the interaction between two spheres, one of which is assumed to be infinitely rigid. Given that the cantilever tip can be indeed described as an infinitely rigid body, it is possible to use this equation to describe the force–displacement relationship during the loading phase of an indentation experiment:

1 $$F = \frac{{4E_{{{\text{el}}}} R^{1/2} }}{{3\left( {1 - \nu^{2} } \right)}} \cdot \delta^{3/2}$$

$\delta$ is the indentation, *E*_el_ is the Young’s modulus of the cytoplasm, $\nu$ is the Poisson’s ratio and $R$ is the equivalent radius calculated using Eq. (2):

2 $$R\; = \;\left( {\frac{1}{{R_{{{\text{cell}}}} }}\; + \; \frac{1}{{R_{{{\text{tip}}}} }}} \right)^{ - 1}$$

where *R*_*cell*_ is the radius of the cell and *R*_*tip*_ is the radius of the cantilever spherical tip.

When introducing the viscoelastic behaviour of the cell (i.e. the stress relaxation phenomenon) the relationship between the force and the displacement could be described with a Solid Linear Standard model (SLS) with a deformation given by the one of the Hertz model, as described in Eq. (3)^9,10^:

3 $$F\; = \;\frac{{4E_{{\text{R}}} R^{1/2} \delta^{3/2} }}{{3\left( {1 - \nu } \right)}} \cdot \left( {1 + \frac{{\tau_{\sigma } - \tau_{\varepsilon } }}{{\tau_{\varepsilon } }}e^{{ - t/\tau_{\varepsilon } }} } \right)$$

where $\tau_{\sigma }$ and $\tau_{\varepsilon }$ are the relaxation times under constant load and constant deformation respectively, while *E*_*R*_ corresponds to the stiffness *k*_*1*_ of the Maxwell’s standard linear solid model (Fig. 3b). By fitting Eq. (3) to a force–displacement curve, it is possible to obtain a standard linear solid representation of the cell’s viscoelastic response, where:

4 $$E_{0} = k_{1} \left( {1 + \frac{{\tau_{\sigma } - \tau_{\varepsilon } }}{{\tau_{\varepsilon } }}} \right) = E_{R} \left( {1 + \frac{{\tau_{\sigma } - \tau_{\varepsilon } }}{{\tau_{\varepsilon } }}} \right)$$

5 $$E_{\infty } = k_{1} \left( {1 + \nu } \right) = E_{R} \left( {1 + \nu } \right)$$

where $E_{0}$ and $E_{\infty }$ are the instantaneous and long-term elastic response respectively and $\nu$ is the Poisson’s coefficient of the cell.^9^

Firstly, the AFM experiment was simulated with a homogeneous computational model, similarly to past works.^1,35,41^ Both a linear elastic and a Neo-Hookean hyperelastic formulations were adopted and compared with respect to the Hertz analytical model, to confirm the almost equal response. In Fig. 3 we reported the comparison between the linear elastic Hertz model and different model configurations all with the Neo-Hookean material formulation.

The force-displacements curves obtained using the two different indenter sizes (namely *R* = 2.5 μm and 5 μm) revealed a similar behaviour of both material formulations, close to the Hertz predictions, thus they can be used as a reference for the comparison with the complex continuum-tensegrity model (Fig. 3a).

In addition, stress relaxation data were obtained from the second step of the simulations, and reported with reference to the Hertz viscoelastic model (Eq. 1) in Fig. 3b.

In order to qualitatively and quantitatively describe the contribution of the cytoplasm versus the cytoskeleton, the mechanical properties of each subcomponents were altered using various parameters combinations, as reported in Table 3. The parameter *Q* = *E*_*el1*_/*E*_*el2*_ = 12.78 is the ratio between the elastic moduli of the cytoplasm of cell type 1 (*E*_*el1*_) and cell type 2 (*E*_*el2*_), and it was used to increase or decrease the mechanical properties between these cell types, obtaining other four sets of mechanical properties (Table 4).

**Figure 4:**
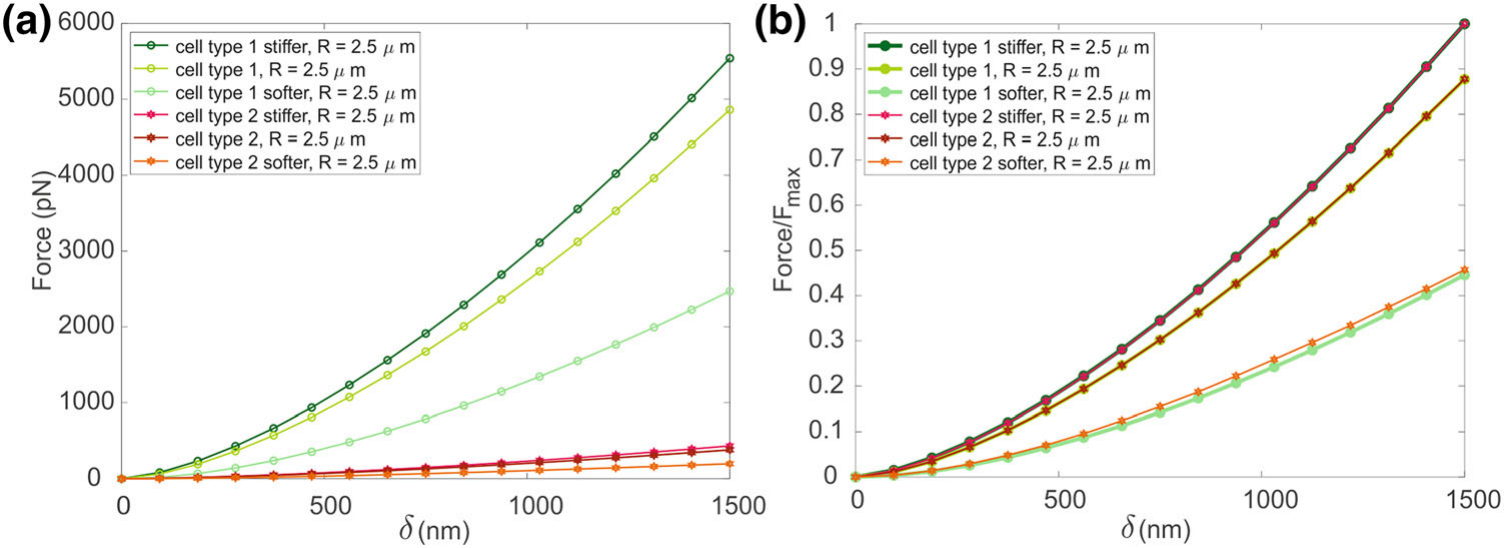
(a) Comparison of different combinations of material properties for the cytoskeleton and the cytoplasm to observe the role of the tensegrity structure in the overall mechanical response of the model. Stiffer refers to the cell type 1 or 2 multiplied by *Q*, while softer states for cell type 1 or 2 divided by *Q*. The parameters of each curve are described in Table 3. (b) normalized results with respect to the maximum force obtained for both cell type 1 and 2.

The results of these simulations are reported in Fig. 4a while normalized values with respect to the maximum force reached in each group is shown in Fig. 4b.

**Table 3:** Mechanical parameters for cell type 1 and 2

		Cytoplasm	Microtubules	Microfilaments	Cell membrane	Nucleus
Fixed parameters	*E*_*r*_ (MPa)	4.50E−04	–	–	–	–
	*τ*_σ_ (s)	19.7	–	–	–	–
	*τ*_ε_ (s)	9.5	–	–	–	–
	*ν* (–)	0.37	0.38	0.38	0.3	0.37
Cell type 1	*E*_*el*_ (MPa)	1.28E−03	1.53E+04	3.32E+04	1.28E−02	5.11E−03
	*C*_*10*_ (MPa)	2.33E−04	2.78E+03	6.02E+03	2.46E−03	9.33E−04
	*D*_*1*_ (MPa^−1^)	1.22E+03	9.39E−05	4.33E−05	1.88E+02	3.05E+02
Cell type 2	*E*_*el*_ (MPa)	1.00E−04	1.20E+03	2.60E+03	1.00E−03	4.00E−04
	*C*_*10*_ (MPa)	1.83E−05	2.17E+02	4.71E+02	1.92E−04	7.30E−05
	*D*_*1*_ (MPa^−1^)	1.56E+04	1.20E−03	5.54E−04	2.40E+03	3.90E+03

These values were used to evaluate the role of the tensegrity structure with respect to the overall mechanical response of the computational model

It is possible to observe how the overall mechanical response is more affected by a decrease in the mechanical properties of the cytoskeleton rather than by its increase.

### Micropipette Aspiration (MPA)

In order to represent the mechanical behaviour of a cell during micropipette aspiration, it is customary to display the displacement of the cell during the application of a constant negative pressure inside the chamber of the micropipette. The half-space viscoelastic model frequently used to represent the evolution of the displacement of the cell has been developed by Sato *et al*.^34^ which represents an extension of the previous half space elastic model by Theret *et al*.^37^ This viscoelastic model assumes an incompressible behaviour; thus, the aspiration length is obtained from the following equation:

6 $$L_{{\text{p}}} \left( t \right)\; = \;R_{p} \frac{\phi \Delta P}{{\pi E_{1} }}\left( {1 - \frac{{E_{2} }}{{E_{1} + E_{2} }}e^{ - t/\tau } } \right)$$

where $L_{p}$ is the projection length of the cell inside the pipette, $R_{p}$ is the micropipette radius, ${\Delta }P$ is the pressure applied to the cell’s surface, $E_{2}$ and $E_{1}$ are the elastic constants of the springs of the corresponding Maxwell standard linear solid representation of the cell, $\tau$ is the characteristic time of the creep response and finally $\phi$ is the “punch coefficient” usually reported to be $\phi \approx 2.1$.^19,41^

However, the half-space analytical model presents some limitations when applied to the cell biomechanics, as also already reported in some works,^1,41^ since cell behaviour is compressible, with an average Poisson’s coefficient of 0.35–0.4 and strains are not infinitesimal.

**Figure 5:**
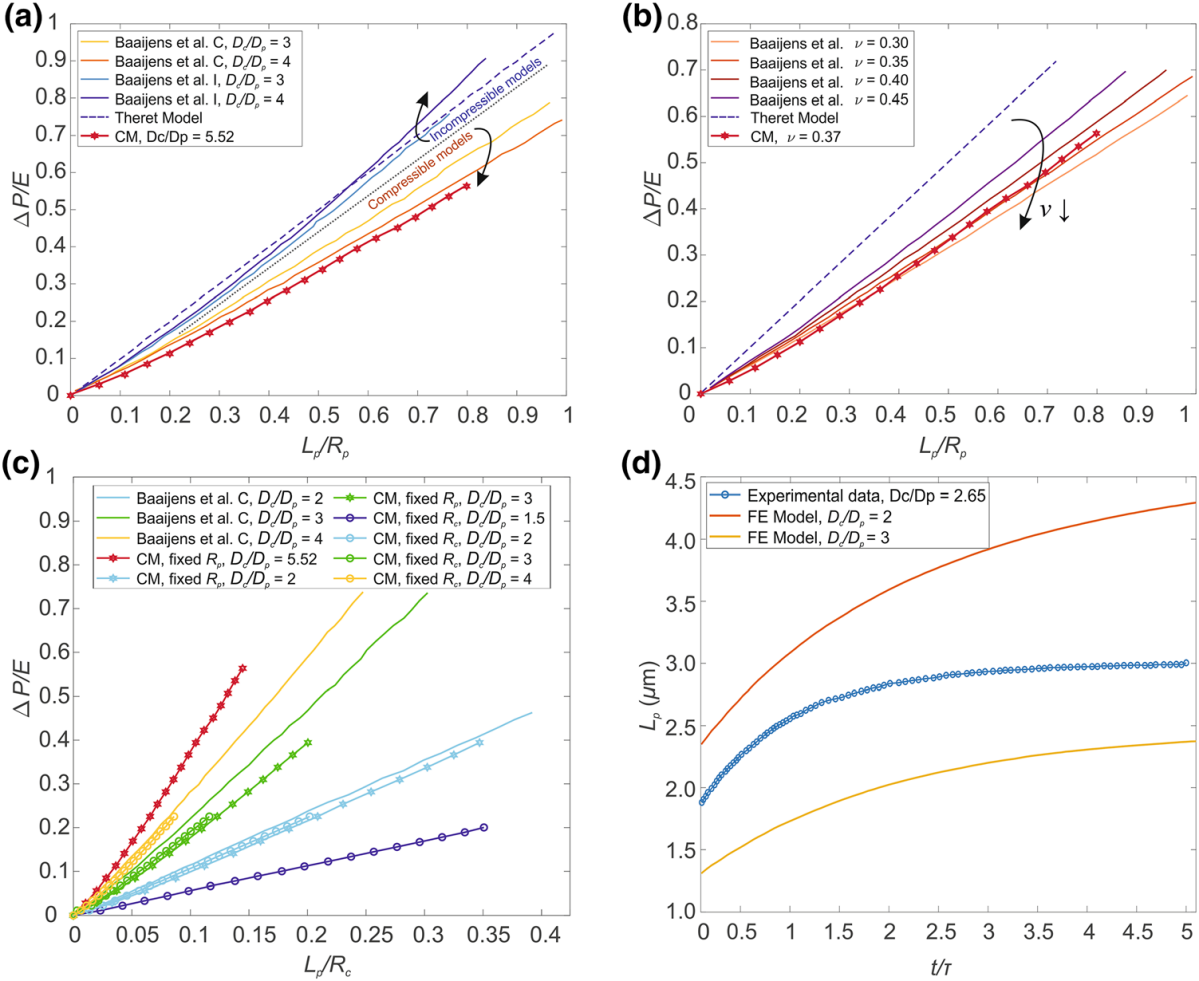
(a) Comparison between incompressible (I) and compressible (C) models of the loading phase of a cell undergoing micropipette aspiration for different values of *D*_*c*_*/D*_*p*_. The curves from the work of Baaijens *et al*.^25^ are compared to the Half-Space analytical model (dashed line) and to our simulation (red line with stars). The cell diameter is fixed. (b) Dependence of the model by the Poisson’s ratio. Data from the work of Baaijens *et al*.^25^ are reported, compared with our simulation (red line with stars), and the Half-Space model with a dashed blue line. (c) Comparison of simulations of the loading phase of the micropipette aspiration employing different *D*_*c*_*/D*_*p*_ ratios. Different colors highlight the ratios (blue for 1.5, light blue for 2, green for 3, yellow for 4 and red for 5.52) while different styles were used to identify our data and Baaijens *et al*.^25^ Data are normalized with respect to cell radius instead of micropipette radius. (d) Comparison between experimental data (from Baaijens *et al*.^25^) and simulations by changing *D*_*c*_*/D*_*p*_.

In Fig. 5a it is possible to observe the comparison between compressible and incompressible models of the micropipette aspiration during the loading phase using different values of the ratio between the micropipette diameter and the cell diameter. Some curves are obtained from a work of Baaijens *et al*.^1^ and are compared with our simulations and the half-space model developed by Theret *et al*.^37^

In Fig. 5b it t is possible to observe the influence of the Poisson’s ratio during the loading phase, comparing our model with different results by Baaijens *et al*.^1^ and the half-space model, highlighting the effects of adopting a compressible model instead of an incompressible one.

Since the ratio *D*_*c*_/*D*_*p*_ has shown to play a significant role on the resulting stimulus–response curves, different ratios have been further investigated (Fig. 5c) it and compared with previous results obtained from literature.^1^ To account for possible coupling and scale effects, both the micropipette and the cell radii were alternatively changed, resulting in different combinations of *D*_*c*_/*D*_*p*_ values, as reported in Table 4.

**Table 4:** Mechanical properties obtained by mixing cell type 1 and 2.

		Cytoplasm	Microtubules	Microfilaments	Cell membrane	Nucleus
Cell type 1 *Q	*E*_*el*_ (MPa)	1.28E−03	1.96E+05	4.25E+05	1.63E−01	6.53E−02
	*C*_*10*_ (MPa)	2.33E−04	3.55E+04	7.69E+04	3.14E−02	1.19E−02
	*D*_*1*_ (MPa^−1^)	1.22E+03	7.35E−06	3.39E−06	1.47E+01	2.39E+01
Cell type 1/*Q*	*E*_*el*_ (MPa)	1.28E−03	1.20E+03	2.60E+03	1.00E−03	4.00E−04
	*C*_*10*_ (MPa)	2.33E−04	2.18E+02	4.71E+02	1.92E−04	7.30E−05
	*D*_*1*_ (MPa^−1^)	1.22E+03	1.20E−03	5.50E−04	2.40E+03	3.90E+03
Cell type 2 *Q	*E*_*el*_ (MPa)	1.00E−04	1.53E+04	3.32E+04	1.28E−02	5.11E−03
	*C*_*10*_ (MPa)	1.82E−05	2.77E+03	6.02E+03	2.45E−03	9.33E−04
	*D*_*1*_ (MPa^−1^)	1.56E+04	9.39E−05	4.33E−05	1.88E+02	3.05E+02
Cell type 2/*Q*	*E*_*el*_ (MPa)	1.00E−04	9.39E+01	2.03E+02	7.82E−05	3.13E−05
	*C*_*10*_ (MPa)	1.82E−05	1.70E+01	3.69E+01	1.50E−05	5.71E−06
	*D*_*1*_ (MPa^−1^)	1.56E+04	1.53E−02	7.08E−03	3.07E+04	5.00E+04

Four parameters combinations to stress both the role of the cytoplasm and the cytoskeleton to the overall mechanical cell response

**Figure 6:**
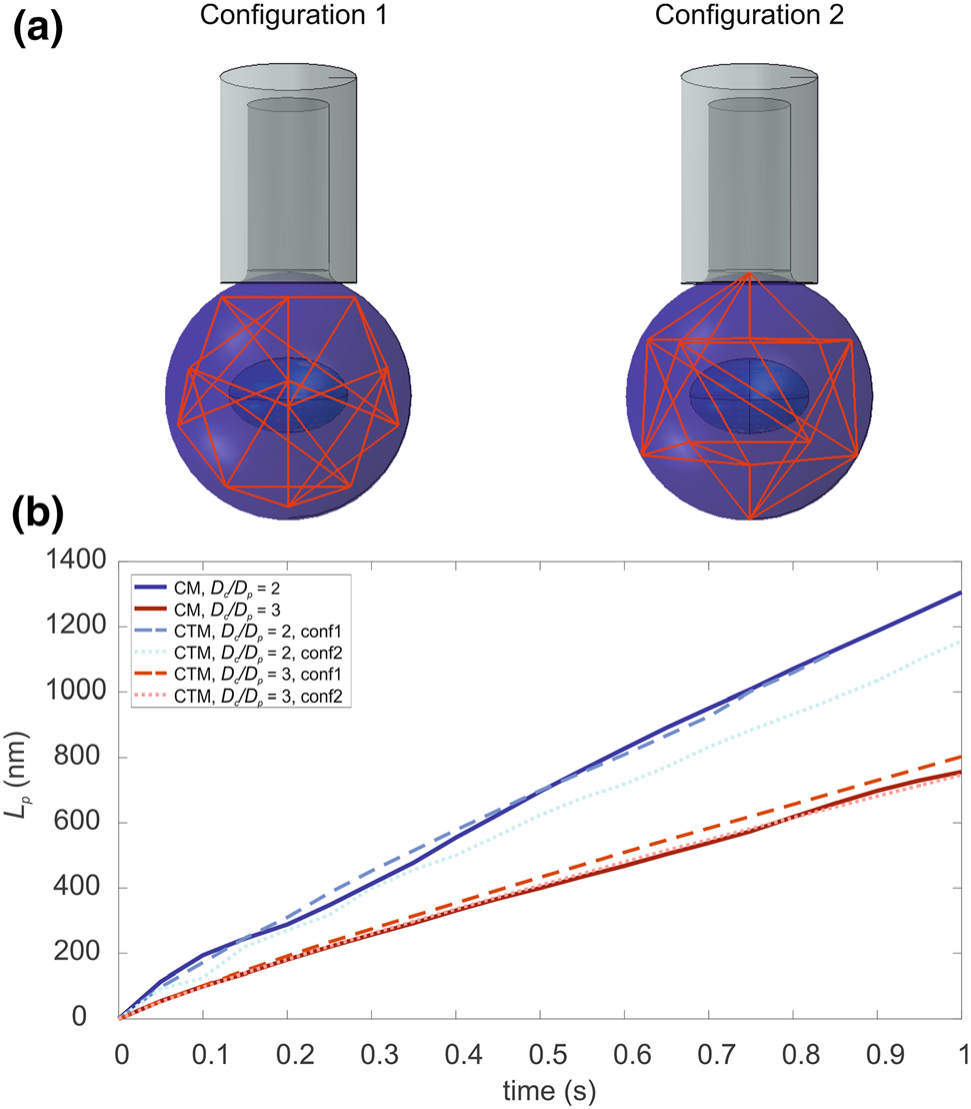
(a) MPA on CTM with two analyzed configurations (1 and 2). (b) Aspiration length in time, with respect to the initial configuration of the cell cytoskeleton. A comparison with the CM is reported, as well as two different case studies with *D*_*c*_/*D*_*p*_ equal to 2 and 3.

The computational model has been tested for its viscoelastic response as well. Two of the simulations obtained using two values of the *D*_*c*_/*D*_*p*_ ratio, as well as the experimental curve from the work of Baaijens *et al*.^1^ are reported in Fig. 5d. Then, once the CM has been validated by comparison with both computational and experimental data obtained from literature, the CTM has been included within the finite element simulation of micropipette aspiration, and compared with respect to the former model. Different orientations of the cytoskeleton could alter the results; thus, two possible tensegrity icosahedron dispositions were used. Figure 6 reported the two analysed configurations and summarizes the results obtained during the loading phase in these simulations.

## Discussion

It has been assessed that a tumour cell exhibits a variation in its mechanical behaviour with respect to a healthy cell, which implies also a change in the mechanotransduction of signals that regulate its normal routine processes. For this reason, in-silico computational models have been developed throughout the years to deepen the knowledge about cell mechanical behaviour without experimental uncertainties. With this aim, in this work we developed a finite element model of a cell and its subcomponents in order to evaluate and quantify the influence of each part, when the same cell is subjected to compression and tensile mechanical stimuli.

The first approach consisted in adopting a continuum model (CM) with a homogeneous material to simulate AFM indentation on a single cell and the related stress relaxation behaviour. Both the loading and stress-relaxation phases were confirmed by Hertz model (Fig. 3), where an optimal correspondence was reached. When the tensegrity structure and the other components were added to the computational model (CTM, Fig. 1), a similar trend was noticed, but characterized by larger values, especially when increasing the indenter size. In particular, the load–displacement curve of the CTM slightly deviated from both the linear Hertz model and the CM when the indenter tip was greater. This aspect highlighted the contribution of the cell subcomponents with respect to the CM, when the behaviour became non-linear (greater displacements). Moreover, these effects became more evident if other parameters combinations were considered, as shown in Fig. 4, where the Young’s modulus of the cytoplasm was kept constant and the other subcomponents’ elastic moduli were variated by a factor *Q*. Computational results showed that a stiffer cytoskeleton, membrane and nucleus contribute in enhancing the response of the cell subjected to a compression force, but this influence is even more noticeable when these subcomponents are characterized by a softer behaviour, which strongly affects the overall cell mechanics (Fig. 4a). Similar trends were found by changing cell type from 1 to 2, which consists of a softer cytoplasm (one order of magnitude lower). When considering the normalized results in order to analyse only the influence of cell subcomponents ((Fig. 4b), it is possible to infer that they significantly modify a cell mechanical behaviour, regardless of the cytoplasm. In Barreto *et al*.^3^ the importance of actin filaments and microtubules during cell compression was highlighted, and in Katti *et al*.^24^ they observed that a decrease in the mechanical properties of the cytoskeleton dramatically influences the global cell behaviour. In addition, from Khunsaraki *et al*.^25^, they observed that the cytoskeleton is the most involved part to carry the reaction force during the AFM tip indentation. From our insights, we can also state that cytoplasm is able to influence the global mechanical response of a cell undergoing AFM indentation, since its variation led to significant different behaviours of the cell (Fig. 4), but cell subcomponents (especially the cytoskeleton) are the ones able to tune the overall cell behaviour in a not uniform way. This is evident in Figs. 4a and 4b, where different mechanical parameters combinations were used: by reducing the mechanical properties of the subcomponents of one order of magnitude, the mechanical response of the cell significantly decreases, while increasing them of the same amount does not influence the global response in the same way.

When dealing with MPA, other useful aspects emerged from the computational modelling. Theret’s elastic model^37^ has been adopted through the years to obtain the elastic properties of the cell (i.e., the elastic modulus) by describing a linear dependence between the normalized aspiration length and the applied pressure (e.g., Refs. 10, 31, 34, 36), as reported by Eq. (6). Theret’s model assumes the cell as a homogeneous incompressible half space and considers only infinitesimal strains. However, it has been observed^1^ that this common approach is not suitable for cells characterized by a Poisson’s coefficient *ν* < 0.5 and subjected to large elongations. Moreover, in MPA, cells and micropipettes sizes are comparable, thus contrasting the hypothesis of the half space model.

Theret’s solution is reported in Figs. 5a, 5b with a dashed blue line, in contrast with our continuum model and previous studies by Baaijens *et al*.^1^. When a compressible material is adopted, it emerges the need of a computational approach to catch the cell biomechanics during MPA. In agreement with Baaijens’ predictions, our results for a single cell, modelled with only cytoplasm as a homogeneous compressible material, revealed a non-linear variation of the aspiration length (*L*_*p*_) when increasing the applied negative pressure.

The influence of cell and micropipette diameters (*D*_*c*_ and *D*_*p*_ respectively) was also investigated, by varying one or both in the range *D*_c_*/D*_p_ from 1.5 to 5.5 (Fig. 5c). As reported in previous works,^1,41^ differences in problem size (i.e., the ratio *D*_*c*_*/D*_*p*_) appear to strongly modified *L*_*p*_; results also highlighted that the parameter which governs this behaviour is not only *D*_*c*_ or *D*_*p*_, but the ratio *D*_*c*_*/D*_*p*_. Figure 5c reports the aspiration lengths of different combinations of *D*_*c*_*/D*_*p*_, normalized by the cell radius instead of the micropipette one, since it usually may vary between cells. This visualization allows to clearly identify the differences by varying *D*_*c*_*/D*_*p*_, in agreement with other proposed models in literature.

When the CTM was used also to model MPA, in order to evaluate possible variations in the final *L*_*p,*_ two cytoskeleton orientations were analysed, namely *configuration 1* and *configuration 2* (Fig. 6a). Slight oscillations with respect to the CM were observed between the two configurations, but some more evident changes arose when the ratio *D*_*c*_*/D*_*p*_ decreases, meaning that a larger portion of the cell is subjected to the negative pressure applied by the pipette. These oscillations highlighted that also in the MPA the cytoskeleton plays a role and some configurations (e.g., *configuration 2*) entail a more significant tensegrity structure involvement (dashed blue lines in Fig. 6b), thus supporting the previous insights obtained in the AFM simulations. Moreover, in MPA both CT and CTM were modelled with the same material parameters adopted in the AFM, thus validated, thanks also to the experimental comparison reported in Fig. 5d.

These two applications of the CTM and the obtained results confirmed the great importance and advantages in considering the main cell subcomponents when modelling a single cell subjected to various mechanical stimuli. Even if the here presented model presents some limitations (such as the lack in connections between the nucleus and the other parts or the missing interactions of the cell with the substrate), it represents a step forward in understanding the differences between cell types from a mechanical point of view. In particular, with our model it is possible to explore how a variation in the mechanical properties of a single subcomponent may affect the overall cell behaviour. Moreover, the main benefit in adopting a tensegrity structure is its potentiality in shape adaptation, a key feature to include when dealing with living cells.

In the present work we proposed a computational model to mimic the biomechanical behaviour of living cells, starting from a continuum model, and then adopting a combined continuum-tensegrity approach in order to elucidate how a variation in the parameters of cell subcomponents may alter the global cell response. Being able to model this intrinsic variability is a key factor in cell mechanics, since it is also associated with a different behaviour between healthy and tumour cells. The analysis of the here proposed CM with reference to other past results from the literature confirmed its applicability for these future purposes, while the outcomes of the CTM with respect to the CM underlined the non-negligible mechanical contributes of the cell subcomponents. In particular, the results highlighted that, when a single cell is subjected to AFM indentation and micro-pipette aspiration (MPA) the cytoskeleton may strongly alter the overall cell biomechanics.

Indeed, these FE models represent a useful tool for the mechanical investigation of both living and cancer cells, revealing to be also a valuable steppingstone in the studies of the mechanical processes that undergo during the different stages of tumour cells and to overcame the complexity in studying the neoplasms, in particular when referring to the inter and intra tumour variability.
